# Photoluminescence Sensing of Soluble Lead in Children’s Crayons Using Perovskite Nanocrystal In Situ Growth on an Aluminum Hydroxide Layer

**DOI:** 10.3390/bios13020213

**Published:** 2023-02-01

**Authors:** Chen Zhang, Shuya Wang, Jingwen Jin, Hezhou Luo, Yiru Wang, Xi Chen

**Affiliations:** 1 Institute of Analytical Technology and Smart Instruments, College of Environment and Public Healthy, Xiamen Huaxia University, Xiamen 361024, China; 2 Department of Chemistry and the MOE Key Laboratory of Spectrochemical Analysis & Instrumentation, College of Chemistry and Chemical Engineering, Xiamen University, Xiamen 361005, China; 3 SEPL Quality Inspection Technology Service Co., Ltd., Fuzhou 350000, China

**Keywords:** lead ion, perovskite, fluorescence sensing, aluminum hydroxide, watercolor, crayon

## Abstract

In this study, a fluorescence sensing approach for lead ion (Pb^2+^) was developed using in situ growth of methylamine lead bromine (MAPbBr_3_) perovskite on an aluminum hydroxide (Al(OH)_3_) thin layer. The Al(OH)_3_ thin layer could be obtained on a glass slide by liquid phase deposition and is of a large specific surface area and insoluble in water. After sulfhydryl functionalization, the Al(OH)_3_ thin layer reveals effective adsorption and excellent enrichment ability to Pb^2+^ and is additionally used as the substrate for the in situ growth of lead halogen perovskite. The fluorescence sensing of Pb^2+^ could be realized by the fluorescence intensity of lead halogen perovskite on the Al(OH)_3_ layer. The linear relationship between the fluorescence intensity and the concentration of Pb^2+^ was found in the range from 80 to 1500 mg/kg. The detection limit of Pb^2+^ is found to be 40 mg/kg, which is lower than the maximum permission of lead residue in student products (90 mg/kg) stipulated by the National Standard of the People’s Republic of China (GB21027-2020). After being grinded and pre-treated, soluble lead in watercolor paint and crayon samples can be extracted by the sulfhydryl functionalization Al(OH)_3_ layer, then lead halogen perovskite can be generated in situ on the layer to achieve the fluorescence sensing for the determination of soluble lead in the samples.

## 1. Introduction

Lead pollution has become a global threat to the fitness of ecosystems. Anthropogenic lead emission is mainly produced from the exhaust emission of leaded gasoline and industry manufacture, particularly mining, smelting, lead batteries, and common pigments [1]. Generally, lead enters the human body through foods, water, and respiration, forming stable and biotoxic substances by combining with the biomolecules (such as proteins and enzymes), and finally affects the whole process of life activities [2]. Lead is seriously harmful to the brain development of children, resulting in mental decline, inattention, anti-social behavior, and other problems [3]. Long-term exposure and intake of lead in adults can also cause neurological defects, bone damage, renal function degradation, hypertension, and other diseases [4]. Study results reveal that children absorb lead more easily than adults [5]. It is not uncommon for toys, stationery, and even tableware around children to contain a small amount of lead, which can still stay in the child’s body for a long time and be highly toxic [6]. Generally, the low content of lead in the samples does not appear obviously toxic, which makes it difficult to universally monitor the perniciousness for children in a short period of time [7]. Stationery items such as watercolors and crayons are commonly used and touched by children, and some of the harmful compositions transfer into the child’s body easily by licking or touching with their skin. In the National Standard of the People’s Republic of China (GB21027-2020), the content of Pb^2+^ in watercolor or crayon samples is determined using inductively coupled plasma mass spectrometry (ICP-MS), and the value is limited to lower than 90 mg/kg [8]. Up to now, there have been many reports including the fluorescence [9] or electrochemical [10] approaches applied for the determination of Pb^2+^ in water samples, as well as for the crayon sample by ICP-OES [11]. These methods are sometimes inconvenient in the rapid determination because of their expensive instrument or complex procedure. The development of simple and visual approaches for the rapid determination of Pb^2+^ in watercolor or crayon samples is still necessary to reduce its harm to children from the source.

Thin-film extraction (TFE) combining the sampling process and extraction reveals a great advantage due to its simple operation, high extraction efficiency, and less organic solvent consume [12]. After suitable selection of extraction materials, a large amount of target substances could be extracted using TFE in a relatively short time, which significantly improved the determination sensitivity and analytical efficiency compared with the traditional extraction technologies [13,14]. A large number of research results show that TFE can be widely used in food analysis [15], environmental evaluation, clinical analysis, and other fields in combination with spectroscopy and chromatography [15,16,17,18]. Among the extraction materials, aluminum hydroxide, alumina, and aluminum compounds are commonly used as adsorption materials thanks to their excellent absorbability characteristics and the fact that they are almost insoluble in water. Arciniega et al. [18] used an aluminum hydroxide (Al(OH)_3_) and aluminum phosphate (AlPO_4_) complex to adsorb antigens in a biosystem, which thus served as a vaccine immune adjuvant. When the medium was neutral, the phosphate in the gel is negative and Al(OH)_3_ presents as positive. The charge difference improved the adsorption of antigen and promoted the binding of antigen and antibody under the physiological pH condition. Raheem et al. [19] used alumina and Al(OH)_3_ as adsorbents to remove water in argon, alkanes, and sulfur dioxide. Gong et al. [20] found that fluoride adsorbed on Al(OH)_3_ at a lower pH and its desorption occurred at a higher pH, and Al(OH)_3_ could be used to adsorb hydrogen fluoride impurities in air and fluoride in water [21].

In this study, the sulfydryl functionalized Al(OH)_3_ layer was prepared for the determination of Pb^2+^ by liquid phase deposition. At the same time, the prepared Al(OH)_3_ layer was used as the substrate for in situ growth of methylamine lead bromine (MAPbBr_3_) perovskite, which was generated by the reaction of methylammonium bromide (MABr) and the sulfydryl-captured Pb^2+^. In the determination, Pb^2+^ in samples was captured by the sulfydryl functionalized Al(OH)_3_ layer and in situ growth to be MAPbBr_3_ perovskite. The fluorescence intensity of MAPbBr_3_ perovskite has a linear relationship with the concentration of Pb^2+^. Compared with the perilous work using mesoporous Al_2_O_3_ film [22], a durable Al(OH)_3_ layer can be prepared in a large quantity by the liquid deposition method with a low cost and simpleness, which can effectively reduce the interference of oil in stationery samples such as watercolor paints and crayons.

## 2. Materials and Methods

Materials and apparatus are provided in the Supporting Information (SI).

### 2.1. Preparation of Aluminum Hydroxide Thin Layer and Pb^2+^ Extraction

**Figure 1:**
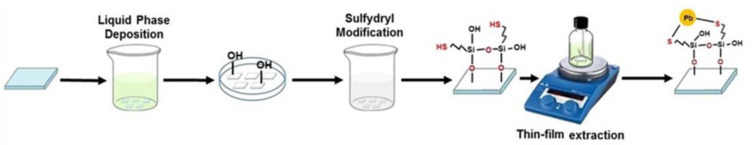
Schematic illustration of Al(OH)_3_-SH layer preparation and Pb^2+^ extraction.

The preparation of the Al(OH)_3_ thin layer by liquid deposition is shown in Figure 1. In the preparation, a glass sheet of 1 cm × 1 cm was successively washed using methanol, acetone, and ultrapure water, and then dried in an oven at 60 °C for used. Then, 2.04 g NaAlO_2_ and 5.76 g urea were dissolved using 200 mL water in a beaker, and the reaction solution was stirred at a speed of 600 rpm at room temperature for 1 h. After this process, the glass sheet was then placed into the beaker for the surface deposition of Al(OH)_3_. The sheet was finally taken out after reaching a constant temperature at 37 °C for 24 h. A white layer on the glass sheet could be observed after the deposition following the below reaction processes (Formulas (1) and (2)):

CO(NH_2_)_2_ + H_2_O → CO_2_↑ + 2NH_3_↑ (1)

NaAlO_2_ + CO_2_ + 2H_2_O → Al(OH)_3_↓ + NaHCO_3_ (2)

The glass sheet deposited with Al(OH)_3_ was washed with ethanol and ultrapure water three times and dried in an oven of 60 °C for use.

In the preparation of the functionalized sulfhydryl Al(OH)_3_ layer, the glass sheet with the Al(OH)_3_ layer was placed into an ethyl acetate solution containing 1~5 wt% MPTS, which was used as a silane coupling agent, and then sealed at room temperature for 24 h. The functionalized layer (Al(OH)_3_-SH) was washed using ultra-pure water and dried at 60 °C for use. In the enrichment of Pb^2+^, as shown in Figure 1, after being thiol functionalized, Al(OH)_3_-SH was used to extract Pb^2+^ in the sample solution. The extraction conditions were set as follows: extraction temperature at 40 °C, extraction time of 15 min, and stirring speed at 800 rpm. After extraction, the glass sheet was taken out, washed with ultra-pure water, and dried in a drying oven at 60 °C.

### 2.2. In Situ Growth of MAPbBr_3_ Perovskite on the Al(OH)_3_-SH Layer Extracted Pb^2+^

The in situ growth of MAPbBr_3_ perovskite was performed using the Al(OH)_3_-SH layer extracted Pb^2+^ by dropping 20 μL of 2.0 g/L MABr (DMF solution), then the glass sheet was dried at 60 °C. As shown in Formula 3, MAPbBr_3_ perovskite could be generated in situ on the layer surface when MABr reacts with Pb^2+^ extracted by Al(OH)_3_-SH, and resulted in green fluorescence emission.

Pb^2+^ + 3MABr → MAPbBr_3_ + 2MA^+^ (3)

### 2.3. Sample Preparation

The process of the sample preparation is referred to in a previous report [23]. Here, 0.0500 g of crushed crayon/watercolor pigment samples were placed into a 50 mL centrifuge tube, 20 mL of 0.1 mol/L HCl was added, and then it was ultrasonically concussed for 1 h. After being filtered, the filtrate was adjusted to pH 7.0 using about 21.5 mL of 0.1 mol/L THAM buffer solution, and then to 50 mL using ultrapure water.

## 3. Results and Discussion

### 3.1. Surface Characterization of Al(OH)_3_ Layer

**Figure 2:**
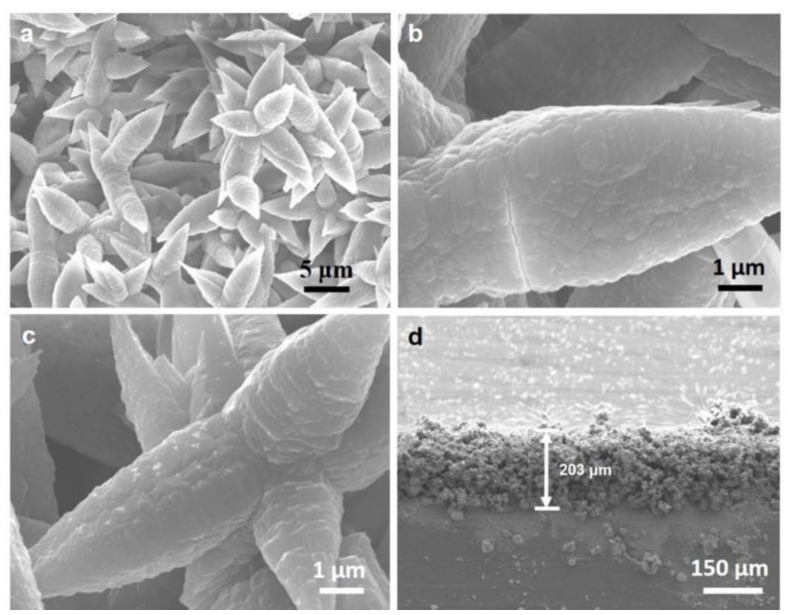
(**a**,**b**) SEM image of the Al(OH)_3_ layer, (**c**) on-site MAPbBr_3_ perovskite on the Al(OH)_3_ layer, and (**d**) section view of the Al(OH)_3_ layer.

The surface morphology of the Al(OH)_3_ layer was characterized using SEM, and the results are shown in Figure 2. Clearly, the spinous flower cluster structure of the Al(OH)_3_ layer provides a larger surface area, and results in more binding sites for the sulfhydryl functionalization and the extraction of Pb^2+^ in the sample solution. In Figure 2b, the uneven surfaces with micro-pores in the spinous flower clusters of Al(OH)_3_ layer provide a suitable substrate for the in situ growth of MAPbBr_3_ perovskite (as shown in Figure 2c), which generates stable fluorescence emission in Pb^2+^ sensing. The thickness of the layer of about 203 μm (Figure 2d) ensures the extraction capacity for Pb^2+^.

**Figure 3:**
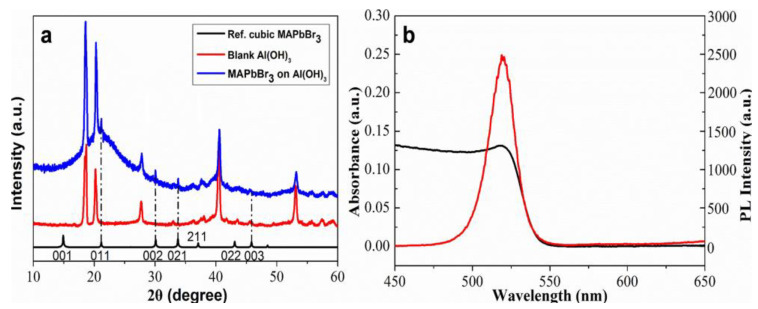
(**a**) X-ray diffraction pattern of MAPbBr_3_ perovskite on-site grown on the Al(OH)_3_-SH layer and (**b**) the absorbance and emission spectra of synthesized MAPbBr_3_ perovskite.

The crystalline structures of aluminum hydroxide generally include α-, β-, and β′-Al(OH)_3_, as well as α-, α′-, and β-AlOOH. XRD was used to characterize the aluminum hydroxide layer. The XRD results as shown in Figure 3 reveal the β-Al(OH)_3_ characteristic diffraction peaks at 2θ 18.879°, 20.514°, and 40.834°, confirming that β-Al(OH)_3_ was obtained in the liquid deposition. Compared with the XRD pattern of MAPbBr_3_ perovskite prepared by thermal injection [24], as seen in Figure 3a, the MAPbBr_3_ perovskite grown in situ on Al(OH)_3_ gives diffraction peaks at (011), (002), (021), and (003), although their intensities are not so strong because of their low content in Al(OH)_3_. In addition, as indicated in Figure 3b, the absorption peak and fluorescence emission peak of MAPbBr_3_ perovskite were found at 518 nm, and 527 nm, respectively.

### 3.2. In Situ Growth of MAPbBr_3_ Perovskite on Al(OH)_3_ Layer

In this work, the Al(OH)_3_ thin layer was prepared and modified using MPTS as a silane coupling agent because of the strong bond effect of S and Pb. In order to obtain the best sensing sensitivity, the preparation conditions for the Al(OH)_3_ layer were optimized. Although the Al(OH)_3_ layer obtained by the liquid deposition method has a large specific surface area, the interaction force between Al(OH)_3_ and Pb^2+^ is not so strong. The introduction of the thiol functionalized reagent to modify the surface of Al(OH)_3_ based on the generation of Pb-S [2] is helpful to enhance the extraction ability of the Al(OH)_3_ layer towards Pb^2+^. As shown in Figure S2a, the blank Al(OH)_3_ layer could only extract a very small amount of Pb^2+^, resulting in the undetectable fluorescence signal because of a low content of MAPbBr_3_ perovskite produced. However, after being modified by MPTS, the fluorescence intensity from the Al(OH)_3_-SH layer obviously increased in 1 mg/L Pb^2+^ solution, and the intensity reached the maximum value as soon as the concentration of MPTS increased to 2.8%. In the experiment, 3% MPTS was selected.

The other extraction conditions such as extraction temperature and time, as well as the stirring rate in the extraction, were optimized. As shown in Figure S2d, with the increase in extraction temperature, the extraction efficiency towards Pb^2+^ of the Al(OH)_3_-SH layer increased, and its efficiency reversely decreased when the temperature was over 50 °C. Hence, the extraction temperature of 40 °C was used. In general, the longer extraction time should enhance the extraction efficiency, and resulted in a higher sensing sensitivity. However, too long an extraction time obviously decreased the analytical efficiency. As indicated in Figure S2c, a suitable extraction time of 15 min was selected. In the extraction process using the Al(OH)_3_-SH layer, the stirring helps to enhance the extraction efficient and shorten the extraction balance time by increasing the substance exchange. Therefore, the influence of the stirring rate on the Al(OH)_3_-SH layer extraction process was investigated. Pb^2+^ in aqueous solution was extracted under the conditions of the stirring rate of 200, 400, 600, 800, 1200, and 1400 rpm, respectively. Fluorescence intensity at different stirring rates is shown in Figure S2b, which indicates that the extraction efficiency reached the best one at the stirring rate of 800 rpm. When the stirring rate was higher than 1000 rpm, the extraction efficiency decreased reversely because of the liquid surface vortex on the layer surface, In addition, the stirring bar jump caused by a high rotational speed is the another factor. In the experiment, the optimal stirring rate was set at 800 rpm.

A suitable medium pH is another important factor for the extraction of Pb^2+^ because of the amphoteric characteristic of Al(OH)_3_. Higher or lower medium pH will cause structural damage to the Al(OH)_3_ layer, resulting in a lower fluorescence intensity. As shown in Figure S2e, a suitable pH in the range from 6.5 to 7.5 could be selected. When pH was beyond the range, the fluorescence intensity significantly decreased because Al(OH)_3_ is converted into Al^3+^ or AlO_2_^−^ under an acidic or alkaline condition, which decreases the extraction efficiency. In the Pb^2+^ sensing, MABr was dropped on the Al(OH)_3_-SH layer, and a product, MAPbBr_3_ perovskite with 527 nm fluorescence emission, could be obtained. Generally, the fluorescence intensity relates to the concentration of Pb^2+^ in sample solutions. The low energy in the production of MAPbBr_3_ perovskite [25] also ensures the sensing response time. As shown in Figure S2f, as the concentration of MABr increased, the amount of in situ generation of MAPbBr_3_ perovskite gradually increased, and resulted in an increase in fluorescence intensity. The best concentration of MABr was found to be 2000 mg/L.

### 3.3. Fluorescence Sensing of Pb^2+^ Using Al(OH)_3_-SH

**Figure 4:**
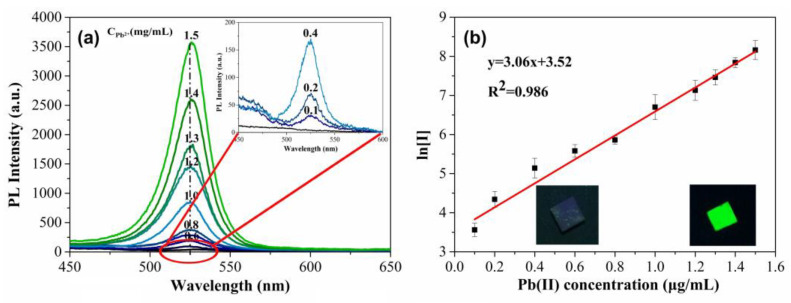
(**a**) PL spectra with different concentrations of Pb^2+^, (**b**) linear relationship between the ln[I] and Pb^2+^ concentration. Insert photographs show the turn-on green PL along with the increase in Pb^2+^ concentration under 365 nm UV excitation.

In the experiment, 0.1 mol/L Pb^2+^ stock solution was diluted to a suitable concentration, and the solution pH was modified to 7.0 using trihydroxymethyl aminomethane buffer solution. After Pb^2+^ in the sample solution is extracted onto the surface of the Al(OH)_3_-SH layer, as indicated in Figure 1, MAPbBr_3_ perovskite grows in situ on the layer surface as soon as the MABr solution is dropped. Obviously, as shown in Figure 4, a higher concentration of Pb^2+^ in solution means more Pb^2+^ is enriched on the Al(OH)_3_-SH layer, and more MAPbBr_3_ perovskite could be produced, resulting in the stronger fluorescence emission. Under the excitation of 365 nm, the maximum fluorescence emission wavelength at 527 nm with a half-peak width of 26 nm could be found.

As shown in Figure 4b, the logarithm of the fluorescence intensity at 527 nm showed a good linear relationship with the concentration of Pb^2+^ in the range from 0.1 to 1.5 mg/L with a linear correlation coefficient (R^2^) of 0.986. The detection limit was found to be 4 × 10^−2^ mg/L based on the ratio of signal intensity (S) and noise (N), by which the detection limit was estimated as the value of three times of S/N.

### 3.4. Stability and Selectivity Investigation for the Sensing Layer

Generally, the ionic salt characteristic of MAPbBr_3_ perovskite is highly susceptible to the influence of water vapor and oxygen in the air. The influence leads to the collapse of the crystal structure and causes the fluorescence quenching. The experiment explores the sensing stability of Al(OH)_3_-SH layer under different usage times. As shown in Figure S3, the fluorescence intensity of the sensing layer remained almost constant within 100 min under atmospheric conditions, indicating that the MAPbBr_3_ perovskite grown in situ Al(OH)_3_-SH layer remains stable for at least 100 min, which ensures the sensing process for Pb^2+^ in sample. MAPbBr_3_ perovskite without template and ligand can remain stable for an acceptable time. It could be speculated that a phenomenon occurs where the hydroxyl groups on the Al(OH)_3_ layer are passivated by sulfydryl functionalization during the surface modification. MPTS acts as the surface ligand to protect MAPbBr_3_ perovskite growing on the Al(OH)_3_ layer from damage by oxygen and water vapor in air [26,27].

In order to investigate the selectivity of the sensing approach, several cations that may affect the extraction ability of Al(OH)_3_-SH layer for Pb^2+^ or co-exist in watercolor paint and crayon samples such as Cr^3+^, Cd^2+^, Ba^2+^, Sb^3+^, and Ag^+^ were selected. As the results show in Figure S4a, using the Al(OH)_3_-SH sensing layer, no cation but only Pb^2+^ could be enriched and reacted on MABr to produce MAPbBr_3_ perovskite to achieve bright fluorescence emission. Although, Ag^+^ will bind with the –SH groups on the Al(OH)_3_-SH layer, which decreased the binding between –SH and Pb^2+^. As shown in Figure S3b, in the same concentration (1 mg/mL) of Ag^+^ and Pb^2+^, the fluorescence intensity decreased to 62% of its original value, respectively. These results reveal that the ordinary metal ions do not generate fluorescence with MABr in the experimental conditions, and the co-existing metal ion with the insoluble characteristics after reaction on –SH, such as Ag^+^, may affect the extraction of Pb^2+^, and results in a decrease in response intensity. Fortunately, there are very low contents of Ag^+^ in stationery samples [28].

### 3.5. Sensing Application for Pb^2+^

In the sensing applications, watercolor paint and crayon samples purchased from the local supermarket were collected, their Pb^2+^ content was analyzed using the proposed sensing approach, and the recovery test was carried out. The test results are shown in Table 1.

Using the sensing approach, the soluble lead content in watercolor paint samples was determined. Seven kinds of watercolor paint samples of different brands and colors were collected. As shown in Table 2, among the collected samples, only one sample (No. 2 sample) with a soluble lead content of 97.7 mg/kg was found, which is slightly over the limit value of the National Standard of the People’s Republic of China (GB21027-2020) [8]. The soluble lead contents in the other six samples were all lower than the detection limit of 80 mg/kg.

## 4. Conclusions

In this study, a sensing approach for the determination of lead content in watercolor paint and crayon samples was developed by in situ extraction of sulfhydryl functionalization aluminum hydroxide substrate. Green fluorescence emission could be obtained with the production of MAPbBr_3_ perovskite on the substrate, by which the lead content in the sample could be determined. The functionalization of sulfhydryl groups on the Al(OH)_3_ layer obviously increases the capture of Pb^2+^ in the sample solution. The MAPbBr_3_ perovskite produced on the Al(OH)_3_-SH layer without ligands is stable because of the channel limited effect [22] of Al(OH)_3_-SH. In addition, compared with the traditional methods for the determination of soluble lead content in watercolor paint and crayon samples, this sensing approach method reveals characteristics of low experimental cost and easier application.

## Figures and Tables

**Table 1:** The determination results of lead in different watercolor samples using the proposed sensing method and the recovery results.

Sample	Before Spiking (mg/kg)	Spiking Level (mg/kg)	Found (mg/kg)	Recovery (%)	RSD (%)
Sample 1	53.5	25	70.8 ± 1.6	90.3	1.7
		50	101.4 ± 3.1	98.0	3.4
		100	162.6 ± 2.3	105.9	5.7
Sample 2	100.7	50	142.7 ± 1.5	94.7	2.1
		100	207.4 ± 2.1	103.1	2.2
		150	275.3 ± 4.4	109.8	3.8

**Table 2:** Determination of soluble lead in crayon samples using the fluorescence sensing method.

Sample	Sensing Method Detected (mg/kg)
Sample 1	^a^N.D.
Sample 2	97.7 ± 7.6
Sample 3	^a^N.D.
Sample 4	^a^N.D.
Sample 5	^a^N.D.
Sample 6	^a^N.D.
Sample 7	^a^N.D.

^a^N.D.: not detected.

## Data Availability

Not applicable.

## Supplementary Materials

The following supporting information can be downloaded at: https://www.mdpi.com/article/10.3390/bios13020213/s1, Figure S1: Schematic illustration of on-site conversion of Pb^2+^ to MAPbBr_3_ perovskite; Figure S2: Effect of (a) the modification quantity of MPTS, (b) stirring rate on extraction procedure, (c) extraction time, (d) extraction temperature, (e) solution pH value and (f) the concentration of supplied MABr on the PL response for Pb^2+^ determination; Figure S3: PL intensity of the MAPbBr_3_ on the Al(OH)_3_ layer versus storage time in air; Figure S4: (a) Fluorescence response of the Al(OH)_3_-SH layer to various metal ions, (b) Fluores-cence response of the Al(OH)_3_-SH layer to the mixture of Pb^2+^ (1 mg/L) and other co-existing cations (1 mg/L).

## References

[1] Flegal A.R., Smith D.R. (1992). Current needs for increased accuracy and precision in measurements of low levels of lead in blood. Environ. Res..

[2] Košak A., Bauman M., Padežnik-Gomilšek J., Lobnik A. (2017). Lead (II) complexation with 3-mercaptopropyl-groups in the surface layer of silica nanoparticles: Sorption, kinetics and EXAFS/XANES study. J. Mol. Liq..

[3] Muntean E., Nicoleta M., Creta C., Marcel D.U.D.A. (2013). Occurrence of lead and cadmium in some baby foods and cereal products. ProEnvironment Promediu.

[4] Zhang C., Wang Y., Cheng X., Xia H., Liang P. (2011). Determination of Cadmium and Lead in Human Teeth Samples Using Dispersive Liquid-liquid Microextraction and Graphite Furnace Atomic Absorption Spectrometry. J. Chin. Chem. Soc..

[5] Abadin H., Ashizawa A., Stevens Y., Llados F., Diamond G., Sage G., Citra M., Quinones A., Bosch S.J., Swarts S.G. (2007). Toxicological Profıle for Lead.

[6] Coco F.L., Monotti P., Cozzi F., Adami G. (2006). Determination of cadmium and lead in fruit juices by stripping chronopotentiometry and comparison of two sample pretreatment procedures. Food Control..

[7] Lidsky T.I., Schneider J.S. (2003). Lead neurotoxicity in children: Basic mechanisms and clinical correlates. Brain.

[8] (2020). Request in Common Use of Security for Student’s Articles.

[9] Cai Y., Ren B., Peng C., Zhang C., Wei X. (2021). Highly Sensitive and Selective Fluorescence “Turn-On” Detection of Pb (II) Based on Fe3O4@Au–FITC Nanocomposite. Molecules.

[10] Zhang C., Lai Z., Liu X., Ye M., Zhang L., Zhang L., Chen X. (2022). Voltammetric determination of Pb^2+^ in water using Mn-doped MoS_2_/MWCNTs/Nafion electrode coupled with an electrochemical flow analysis device. Electroanalysis.

[11] Đogo-Mračević S., Ražić S., Trišić J., Mitrović N., Đukić-Ćosić D. (2022). Toxic elements in children’s crayons and colored pencils: Bioaccessibility assessment. J. Serb. Chem. Soc..

[12] Wilcockson J.B., Gobas F.A. (2001). Thin-film solid-phase extraction to measure fugacities of organic chemicals with low volatility in biological samples. Environ. Sci. Technol..

[13] Bruheim I., Liu X., Pawliszyn J. (2003). Thin-film microextraction. Anal Chem..

[14] Olcer Y.A., Tascon M., Eroglu A.E., Boyacı E. (2019). Thin film microextraction: Towards faster and more sensitive microextraction. TrAC-Trends Anal Chem..

[15] Gómez-Ríos G.A., Gionfriddo E., Poole J., Pawliszyn J. (2017). Ultrafast Screening and Quantitation of Pesticides in Food and Environmental Matrices by Solid-Phase Microextraction-Transmission Mode (SPME-TM) and Direct Analysis in Real Time (DART). Anal Chem..

[16] Piri-Moghadam H., Gionfriddo E., Rodriguez-Lafuente A., Grandy J.J., Lord H.L., Obal T., Pawliszyn J. (2017). Inter-laboratory validation of a thin film microextraction technique for determination of pesticides in surface water samples. Anal Chim. Acta.

[17] Reyes-Garces N., Bojko B., Pawliszyn J. (2014). High throughput quantification of prohibited substances in plasma using thin film solid phase microextraction. J. Chromatogr. A.

[18] Cai L., Dong J., Wang Y., Chen X. (2019). A review of developments and applications of thin-film microextraction coupled to surface-enhanced Raman scattering. Electrophoresis.

[19] Nwosu F.O., Ajala O.J., Owoyemi R.M., Raheem B.G. (2018). Preparation and characterization of adsorbents derived from bentonite and kaolin clays. Appl. Water Sci..

[20] Gong W.X., Qu J.H., Liu R.P., Lan H.C. (2012). Effect of aluminum fluoride complexation on fluoride removal by coagulation. Colloid Surf. A.

[21] Ju J., Liu R., He Z., Liu H., Zhang X., Qu J. (2015). Utilization of aluminum hydroxide waste generated in fluoride adsorption and coagulation processes for adsorptive removal of cadmium ion. Front. Environ. Sci. Eng..

[22] Wang S., Huang Y., Zhang L., Li F., Lin F., Wang Y., Chen X. (2021). Highly selective fluorescence turn-on determination of Pb(II) in Water by in-situ enrichment of Pb(II) and MAPbBr3 perovskite growth in sulfydryl functionalized mesoporous alumina film. Sens. Actuators B-Chem..

[23] Lin G., Chen Q. (2002). The Methodology of Lead Detection and Lead Survey in Stationery for Painting and Writing. Master’s Thesis.

[24] Jaffe A., Lin Y., Beavers C.M., Voss J., Mao W.L., Karunadasa H.I. (2016). High-Pressure Single-Crystal Structures of 3D Lead-Halide Hybrid Perovskites and Pressure Effects on their Electronic and Optical Properties. ACS Cent. Sci..

[25] Oranskaia A., Yin J., Bakr O.M., Brédas J.L., Mohammed O.F. (2018). Halogen Migration in Hybrid Perovskites: The Organic Cation Matters. J. Phys. Chem. Lett..

[26] Li S., Lei D., Ren W., Guo X., Wu S., Zhu Y., Rogach A.L., Chhowalla M., Jen A.K.Y. (2020). Water-resistant perovskite nanodots enable robust two-photon lasing in aqueous environment. Nat. Commun..

[27] Zhang X., Wu X., Liu X., Chen G., Wang Y., Bao J., Xu X., Liu X., Zhang Q., Yu K. (2020). Heterostructural CsPbX(3)-PbS (X = Cl, Br, I) Quantum Dots with Tunable Vis-NIR Dual Emission. J. Am. Chem. Soc..

[28] Rastogi S., Pritzl G. (1996). Migration of some toxic metals from crayons and water colors. Bull. Environ. Contam. Toxicol..

