# Supplementary material for: Photoluminescence Sensing of Soluble Lead in Children’s Crayons Using Perovskite Nanocrystal In Situ Growth on an Aluminum Hydroxide Layer
Source: Biosensors (Basel). 2023 Feb 1;13(2):213. doi: 10.3390/bios13020213 (PMC9954079; doi:10.3390/bios13020213)
Supplement: Supplementary file 1 [file biosensors-13-00213-s001.zip › biosensors-2112601-supplementary.pdf]

biosensors

Supplementary Materials

Photoluminescence Sensing of Soluble Lead in Children's Crayons Using Perovskite Nanocrystal In Situ Growth on an Aluminum Hydroxide Layer

Chen Zhang [1,†], Shuya Wang [2,†], Jingwen Jin [1], Hezhou Luo [3], Yiru Wang [2], and Xi Chen [2,*]

[1] Institute of Analytical Technology and Smart Instruments, College of Environment and Public Healthy, Xiamen Huaxia University, Xiamen 361024, China
[2] Department of Chemistry and the MOE Key Laboratory of Spectrochemical Analysis & Instrumentation, College of Chemistry and Chemical Engineering, Xiamen University, Xiamen 361005, China
[3] SEPL Quality Inspection Technology Service Co., Ltd., Fuzhou 350000, China
* Correspondence: xichen@xmu.edu.cn
† These authors contributed equally to this work.

Materials and methods

1. Materials and chemicals

Sodium aluminate ($NaAlO_2$) and 3-mercaptopropyl triethoxysilane (MPTS) were purchased from Aladdin Reagent Co., Ltd. (Shanghai, China). Lead acetate ($Pb(Ac)_2·3H_2O$), copper chloride ($CuCl_2·2H_2O$), antimony trichloride (SbCl3), barium chlo-ride ($BaCl_2·2H_2O$), mercury sulfate ($HgSO_4$), silver nitrate (Ag NO_3), chromium chloride ($CrCl_3$), cadmium chloride ($CdCl_2·6H_2O$), methylammonium bromide (MABr) and trihy-droxymethyl aminomethane (THAM, $C_4H_{11}NO_3$) used for buffer solution, were obtained from Sinopharm Chemical Reagent Co., Ltd. (Shanghai, China). All of the reagents are analytical grade. The ultra-pure water used in the experiment is prepared by a Millipore ultra-pure water system (18.2 $M·\Omega cm^{-1}$). Pb^{2+} reserve solution was prepared by dissolving lead acetate in 0.1 M HCl solution. The reserve solution was diluted to the desired con-centration of Pb^{2+} during the experiment. Samples of crayons and watercolor pigments were purchased from a local supermarket.

2. Apparatus

An F-7100 fluorescence spectrophotometer (Hitachi, Japan) was used to collect the fluorescence spectra of $MAPbBr_3$ perovskite. In the experiment, the slit width for the excitation and emission was set at 1.0 nm and 2.5 nm, respectively. The time-resolved fluorescence spectra and absolute photoluminescence quantum yield (PLQY) were recorded on a FS5 fluorescence spectrophotometer (Edinburgh, England) equited with a 150 W xenon lamp and a nanosecond flash lamp (365 nm).The morphology of $MAPbBr_3$ perovskite grown in situ was measured using S4800 scanning electron microscopy (SEM, Hitachi, Tokyo, Japan), and its crystal structure was characterized by X-ray diffraction using Ultima IV X-ray Diffractometer (Rigaku, Japan) with an operating voltage of 40 kV and a current of 15 mA. (Cu Kα, 5 kV, 15 mA). An UV-visible spectrometer (UV-2550, Shimadzu Company, Japan) was used to record the of UV-visible spectrum change of MAPbBr3 per-ovskite.

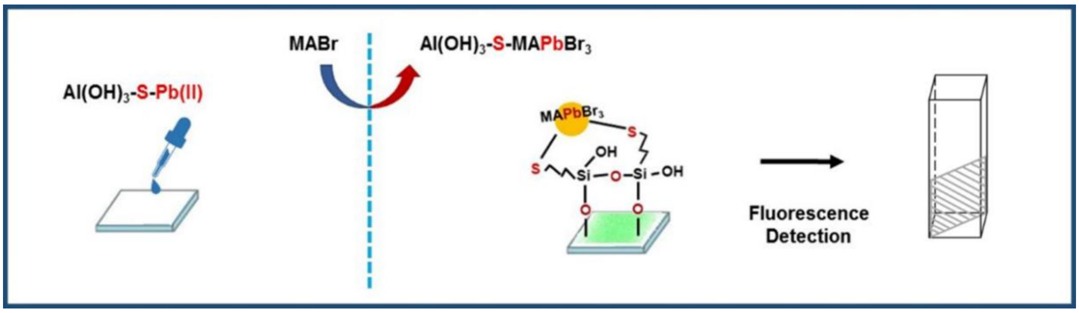

Figure S1. Schematic illustration of on-site conversion of Pb^{2+} to $MAPbBr_3$ perovskite.

In the sensing, the hydroxyl groups on the $Al(OH)_3$ layer surface modified sulfydryl by the hydrolysis of silane. Pb^{2+} in sample was efficiently enriched on the $Al(OH)_3$ layer . The layer was then taken out and dried in an oven at 40°C, followed by the addition of excess MABr solution on the layer. The solvent on the layer was also evaporated in an oven. Upon the drying, $MAPbBr_3$ nanocrystals were formed on the $Al(OH)_3$ layer , and green fluorescence emission of the Product, $MAPbBr_3$ nanocrystals, could be observed under the excitation of 365 nm ultraviolet light. The PL intensity showed linearly relationship with the Pb^{2+} concentration in the sample solution. In the experiments, the different fluorescence intensity caused by different content of Pb (II) was recorded using FL 7100 fluorescence spectrophotometer with the excitation wavelength of 365 nm. The fluorescence intensities of four edges and center of the glass slide with $Al(OH)_3$-SH layer grown with $MAPbBr_3$ perovskite were tested, and the results were averaged. In the determination the concentration of Pb(II), the fluorescence emission wavelength of 527 nm was set. The limit of detection (LOD) of the method was estimated when the fluorescence signal from a certain low concentration of Pb(II) was three times than that of the noise signal generated by the blank sample (S/N = 3).

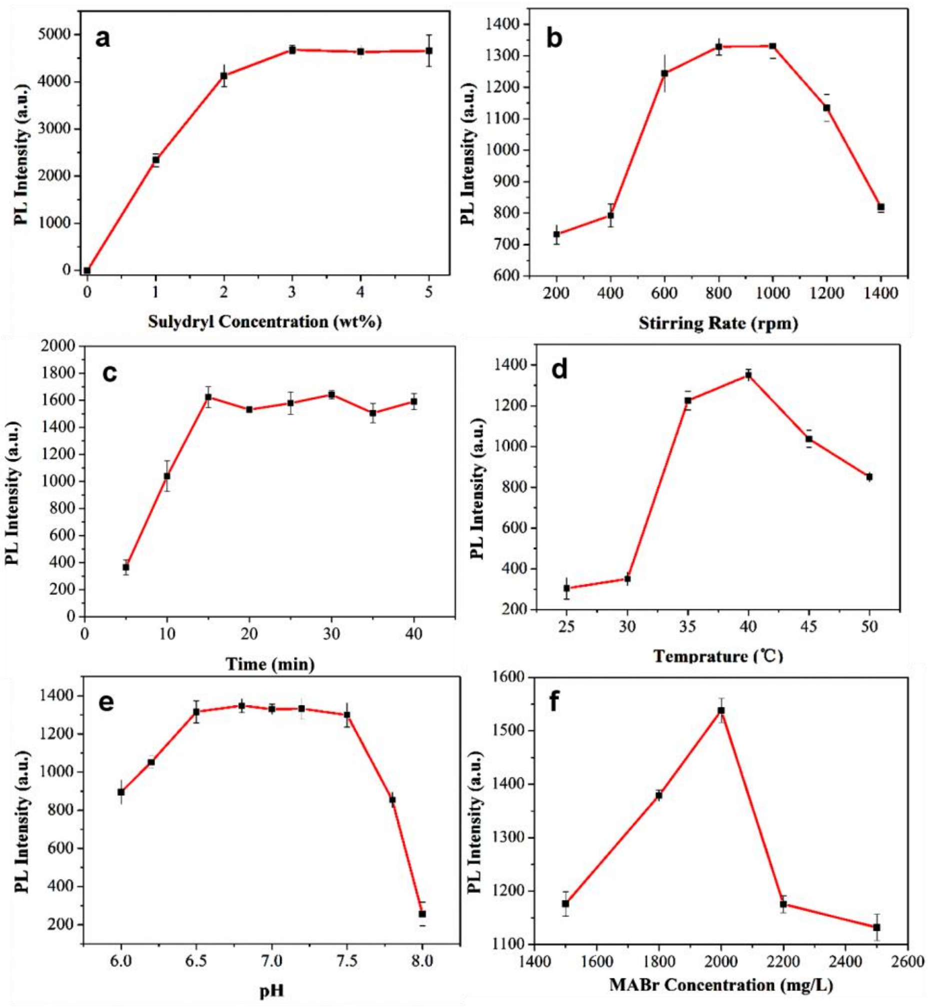

Figure S2. Effect of (a) the modification quantity of MPTS, (b) stirring rate on extraction procedure, (c) extraction time, (d) extraction temperature, (e) solution pH value and (f) the concentration of supplied MABr on the PL response for Pb^{2+} determination.

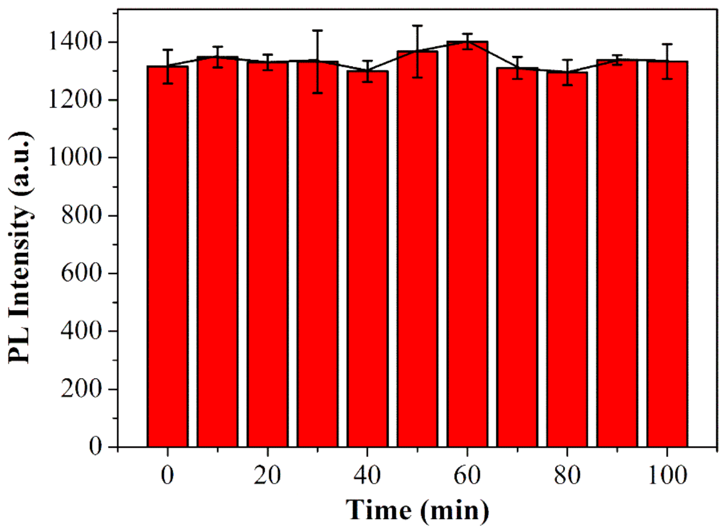

Figure S3. PL intensity of the $MAPbBr_3$ on the $Al(OH)_3$ layer versus storage time in air.

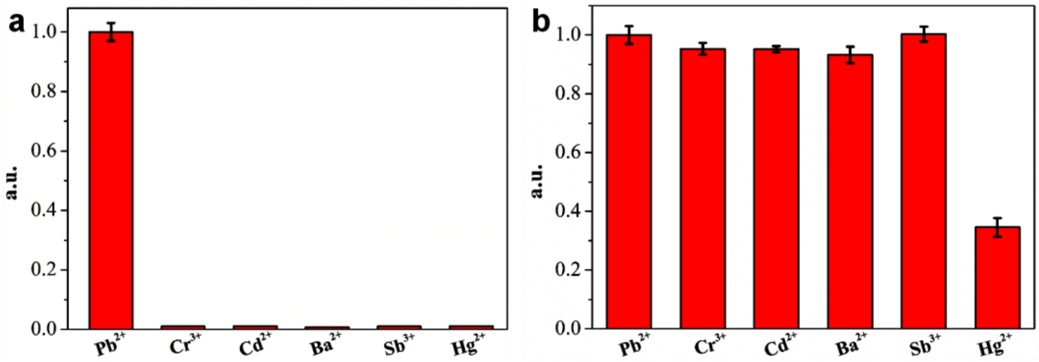

Figure S4. (a) Fluorescence response of the $Al(OH)_3$-SH layer to various metal ions, (b) Fluorescence response of the $Al(OH)_3$-SH layer to the mixture of Pb^{2+} (1 mg/L) and other co-existing cations (1 mg/L).
